# Charge Injection and Dielectric Characteristics of Polyethylene Terephthalate Based on Semiconductor Electrodes

**DOI:** 10.3390/ma14061344

**Published:** 2021-03-10

**Authors:** Guan-Yu Liu, Wei-Feng Sun, Qing-Quan Lei

**Affiliations:** 1 Key Laboratory of Engineering Dielectrics and Its Application, Ministry of Education, School of Electrical and Electronic Engineering, Harbin University of Science and Technology, Harbin 150080, China; lgy_0219@sina.com; 2 College of Materials Science and Engineering, Qingdao University of Science and Technology, Qingdao 266042, China; sunweifeng@hrbust.edu.cn

**Keywords:** charge injection, dielectric performance, semiconductor electrode, charge trap

## Abstract

Employing a novel semiconductor electrode in comparison with the traditional semiconductor electrode made of polyethylene/ethylene-vinyl-acetate copolymer/carbon-black (PE/EVA/CB) composite, characteristic charge carriers are injected into polyethylene terephthalate (PET) as a polymer dielectric paradigm, which will be captured by specific deep traps of electrons and holes. Combined with thermal stimulation current (TSC) experiments and first-principles electronic-state calculations, the injected charges from the novel electrode are characterized, and the corresponding dielectric behavior is elucidated through DC conductance, electrical breakdown and dielectric spectrum tests. TSC experiments with novel and traditional semiconductor electrodes can distinguish the trapping characteristics between hole and electron traps in polymer dielectrics. The observable discrepancy in space charge-limited conductance and the stable dielectric breakdown strength demonstrate that the electron injection into PET film specimen is restricted by using the novel semiconductor electrode. Attributed to the favorable suppression on the inevitable electron injections from metal electrodes, adopting novel i-electrode can avoid the evident abatement of dipole orientation polarization caused by space charge clamp, but will engender the accessional high-frequency dielectric loss from dielectric relaxations of interface charges at i-electrodes.

## 1. Introduction

In electrical power systems of high voltage direct current (HVDC) transmission, polymer materials used for the insulation layer in power transmission cables are susceptible to suffering space charge accumulations that could expedite electrical aging and breakdown-failure process, which leads to an evident abatement in working life and operation stability of HVDC cable [1,2,3]. In recent years, the researches on space charge accumulation in insulation layer of HVDC cable have focused on how to improve dielectric properties of insulating materials such as low-density polyethylene and cross-linked polyethylene. Despite the generally affirmed limitation of charge traps on carrier transports in insulation layer, space charge injection in HVDC mini-cable is closely related to the type of semi-conductive shielding layer [4,5]. Space charge characteristics of insulation layer will greatly depend on the type of charge carriers (electron or hole) injected from specific semi-conductive layer. As an indispensable part of HVDC cable, the semi-conductive shielding layer closely contacting between the insulation layer and conductor core can homogenize the electric field in insulation layer and reduce the space charge injection from metal core. Therefore, it is of great significance to improve the semi-conductive shielding layer for inhibiting charge injections into polymer dielectrics. 

Influence of semi-conductive shielding layer on the space charges accumulated in insulation layer has been revealed by a sandwich structure with the two sides of insulation layer being coated with different semi-conductive materials [6,7]. It has been proved that the effective suppression of space charge accumulation in HVDC cable can be realized by matching the well-optimized semi-conductive materials. Space charge accumulations in cross-linked polyethylene (XLPE) have been studied by using different electrode materials, from the sputtering coated gold to the thermally adhesive semiconductor, which verified that the charge injection and release rely on semiconductor electrodes [8]. Underlying mechanism of semi-conductive layer improving dielectric breakdown strength of XLPE was elucidated by characterizing the thermal diffusion of additives at the interface between semi-conductive and insulating layers with transmission electron microscopy [9,10]. It was proved that glycerol additive could promote the electrical resistance at the interface between semiconductor-electrode and polyethylene, as indicated by the significant increment of electrical breakdown field by more than two times [11]. In particular, the innovative technology of controlling the injected charge-carrier types (electron or hole) by exploiting semiconductor electrodes could lead to a new paradigm of functional polymeric materials such as hole-dominant piezoelectric polymers used for electret transducers [12,13]. 

Traditional semi-conductive shielding layer used in transmission cable is composed of polyethylene/ethylene-vinyl-acetate copolymer (PE/EVA) and carbon-black (CB), on which multiple schemes such as controlling copolymer composition, introducing modifiers, adding fillers, and matching interface have been implemented to ameliorate space charge distributions in insulation layer [14,15,16,17]. The composition of copolymer determines its compatibility with CB, which leads to considerable discrepancy in the aggregation morphology and dispersivity of CB in semi-conductive polymeric composites, and thus affects charge injections at the interface between semi-conductive shielding and insulation layers [18,19]. By introducing fast-ionic conductors into semi-conductive composites, a favorable conductive network could be established to increase the positive temperature coefficient and reduce the kinetic energy of transporting charges, thus improving space charge characteristics of insulation layer [20,21]. Due to the difference of material structures between the composite semiconductor and insulating polymer that should be developed for cable fabrications, pertinent technologies of polymeric hetero-junctions and surface electrodes are urgently desired for constructing the insulation system of transmission cable. Meanwhile, numerical simulation technology can be exploited to reveal the experimentally unattainable attributes such as local electric field distribution, ion thermodynamics, and high-current pulsed surface discharge, which is of great significance to study the charge transport mechanism of dielectrics/semiconductor interfaces [22,23,24].

In the present study, a new type of semiconductor electrode (ionic-electrode, abbreviated as i-electrode in this paper) and the traditional semiconductor electrode made of polyethylene/ethylene-vinyl-acetate copolymer and carbon-black (PE/EVA/CB) composite (electronic-electrode, abbreviated as e-electrode in this paper) are employed to comparatively investigate the charge injection characteristics and the resulted dielectric performances. Polyethylene terephthalate (PET) is chosen as the paradigm dielectric material to be tested with i-electrode or e-electrode for thermally stimulated current (TSC) and electrical conductivity. First-principles electronic-state calculations are performed to analyze charge traps in coordination with TSC tests. Moreover, after applying PET film samples with various levels of voltages by using different semiconductor electrodes, the electrical breakdown experiment and dielectric spectrum test are implemented to explore the acquired insulation performance by semiconductor electrodes and their effects on the dielectric behavior of PET.

## 2. Experimental Schemes

**Figure 1:**
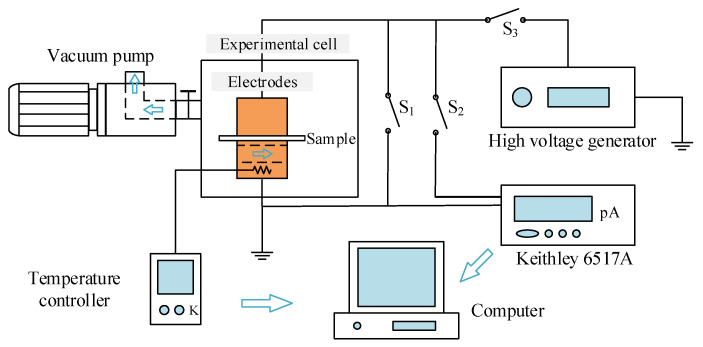
Schematics of TSC test system. S_1_, S_2_, and S_3_ symbolize three individual circuit switches that should be closed for short-circuiting, testing current, and applying voltage, respectively.

**Figure 2:**
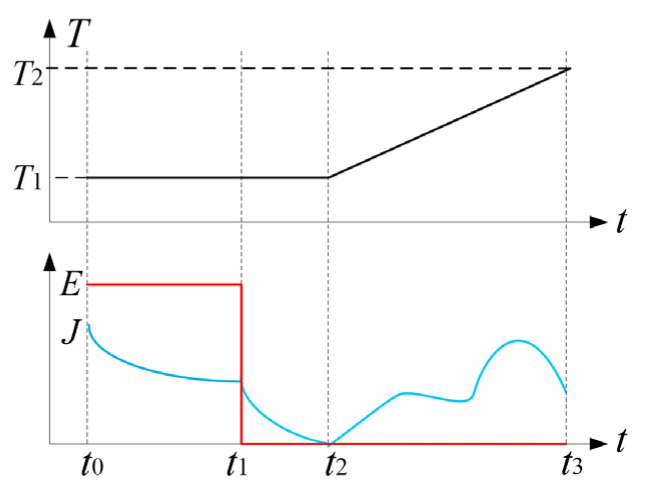
Temperature and electric field strength of PET-electrode system varying with time in TSC test. *E* denotes electric field strength for charge injection; *J* represents electric current; *T*_1_ and *T*_2_ specify the lowest and highest temperatures for measuring TSC, respectively; *t*_0_ and *t*_1_ mark the beginning and end moments of charge injection, respectively; *t*_2_ and *t*_3_ represent the moments when TSC measurement begins and ends, respectively.

In order to investigate the charge injection characteristics of adopting different semiconductor electrodes, TSCs of PET-electrode system are tested to calculate energy level distributions of charge traps that have captured the charge carriers injected from semiconductor electrodes. After being pretreated under short-circuit in a vacuum, drying oven for 24 h to eliminate surface charges, the film PET samples with 50 µm thickness are evaporated with an aluminum electrode (the measuring electrode) on one side and contacted with a semiconductor electrode (the applying-voltage electrode) on the other side. The measurement system of TSC and testing conditions is shown in Figure 1; Figure 2, respectively. Firstly, for charge injection, the electric fields of 25 kV/mm, 50 kV/mm, 75 kV/mm, and 100 kV/mm are individually applied (close switch S_3_) on the PET/electrode testing system for 30 min at ambient temperature, in which carriers are injected from electrodes into PET material and thus being captured by charge traps. After charge injection, the applied voltage is removed out (open switch S_3_), and PET/electrode testing system is short-circuited (close switch S_1_), and then the testing system is promptly cooled down to −20 °C (much lower than *T*_1_–*T*_2_ for measuring TSC) by liquid nitrogen and stabilized for 10 min to “froze” the injected charges in traps. Eventually, by introducing a microcurrent meter into the PET/electrode short-circuit system (open switch S_1_ and close switch S_2_), TSCs are consecutively measured when the testing temperature is gradually raised to 130 °C at a heating rate of 3 °C/min. Since there is no observable TSC arising in testing system at a temperature range between −20 °C and 20 °C, TSC data should be recorded at the temperature range of 20–130 °C.

Electrical conductivity is tested with a standard three-electrode system to avert the interference of leakage currents along sample surface. PET films with 50 µm thickness are fabricated into electrode-testing samples with two aluminum electrodes (as the measuring and protective electrodes respectively) evaporated on one side and one semiconductor electrode for applying a negative high voltage on the other side. The tested samples are applied by various electric fields covering 10‒100 kV/mm range, at each point of which persisting for 60 min to measure conductance current.

PET circular film samples with 50 mm diameter and 50 μm thickness are washed with anhydrous ethanol and then dried in a vacuum oven for 24 h at 100 °C. Furthermore, the dried PET film is evaporated by an aluminum electrode on one side with the other side being coated by an ionic or electronic semiconductor electrode. Subsequently, the electrode-testing samples are polarized under electric fields of 25 kV/mm, 50 kV/mm, 75 kV/mm, and 100 kV/mm individually for 30 min and then dried in a vacuum oven for 30 min before implementing breakdown experiments. Conforming to norm ASTM-D149, DC electrical breakdown fields are measured repeatedly more than 10 times by a voltage-resistant tester (CS2674C, Changsheng Instruments Co. Ltd., Nanjing, China). The maximum voltage is recorded just before the tested sample reaches electrical breakdown by continuously raising the applied electric field with a constant rate of 4 kV/s.

Dielectric spectra of PET samples that have been polarized for 30 min under an electric field of 100 kV/mm with electronic and i-electrodes are tested by a wide frequency dielectric spectrometer (Alpha-A, Novocontrol Co. Ltd., Montabaur, Germany) in combination with a spectrum analyzer (N9320B, Agilent Technologies Co. Ltd., Palo Alto, CA, USA) in the frequency range of 10‒10^6^ Hz at room temperature.

## 3. Results and Discussion

### 3.1. Thermally Stimulated Current

**Figure 3:**
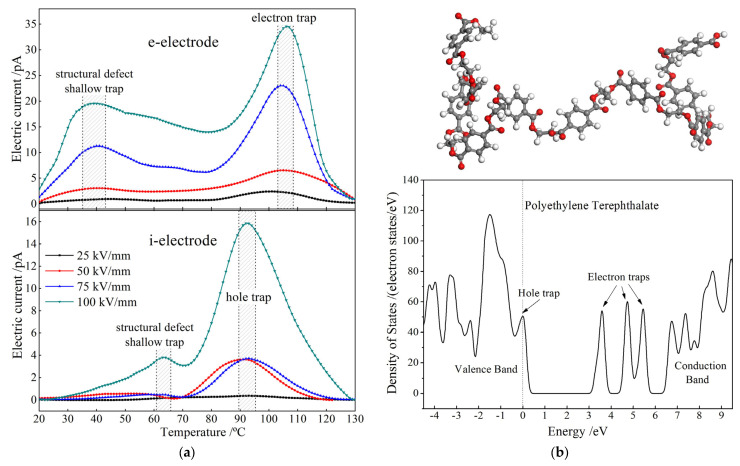
(**a**) TSC temperature spectra tested after charge injections with (**bottom panel**) i-electrode and (**top panel**) e-electrode, respectively, under various electric fields of 25 kV/mm, 50 kV/mm, 75 kV/mm, and 100 kV/mm; (**b**) (**top panel**) the modeled polymeric structure after geometry optimization of PET molecule in 10 polymerizations, and (**bottom panel**) the density of energy states obtained from first-principles electronic-state calculations.

TSC test characterizes electrical currents from the thermally excited charges that have been captured into traps (bound states) of electrons or holes. TSC peak at a specific temperature identifies the density of charge traps with a specific energy level lower than conduction bottom (trap level depth), as shown in Figure 3 illustrating TSC spectra for charge injections from e-electrode and i-electrode, respectively. Since PET is a polar dielectric material with a glass transition temperature of about 88 °C, TSC peaks below and above this temperature originate from the relaxing dipoles of structural defects and the detrapping of space charges, respectively. The general TSC peaks at about 39 °C for e-electrode injection and 62 °C for i-electrode injection derive from the detrapping electrons and holes, respectively, that have been captured into the shallow traps introduced by intrinsic structural defects. At the temperatures of 90–105 °C, both charge injections by e-electrode and i-electrode represent a narrower high-amplitude TSC peak, which originate from the deep traps introduced by polar-groups in PET molecules. More explicit for higher polarization electric field, the observable discrepancy in position and intensity of high-temperature peaks between charge injections by e-electrode and i-electrode implies that they are derived from two types of charge carriers (electron and hole), respectively. Charge carriers injected into polymer dielectrics are more liable to be captured by deep traps than shallow traps, resulting in fewer carriers being trapped into the intrinsic shallow traps of structural defects in PET materials. Therefore, the amplitude and integral area of TSC peaks at about 39 °C and 62 °C are obviously smaller than that at 90–105 °C, which means the charge traps will be gradually occupied from deeper levels to higher levels until to be imbued with the increase of the applied electric field or polarization time, as comparatively shown in Figure 3. Meanwhile, in order to reach the trap-filled limit, the applied electric field is required to approach and even exceed the breakdown value.

Trap densities versus trap level depths (trap level distributions) in PET are calculated from TSC temperature spectra [25] after charge injections with different semiconductor electrodes under various applied electric fields, with the results listed in Table 1. Further, It is consistently verified by the density of electronic states (DOS) obtained from first-principles calculations (as shown in Figure 3b) that the bound states for trapping electrons and holes appear at the energy levels of 1.12 eV and 0.94 eV, referencing to conduction band minimum and valence band maximum, respectively, which coincide with characteristic TSC peaks at 105 °C (1.09 eV) and 90 °C (0.97 eV) presented by the charge injections of e-electrode and i-electrode, respectively, as comparatively shown by the bottom results in Table 1. The polymeric molecule modeling and the first-principles DOS calculations are implemented with the schemes conforming to references [26,27]. In addition, the two deeper traps of 1.85 eV and 2.99 eV deriving from the chemical species in PET molecule cannot be characterized by TSC method because the thermal stimulation temperature required for detrapping charges from such deep traps will approach >300 °C, which have distinctly exceeded the melting point of PET material.

### 3.2. Electrical Conductance

Generally, for analyzing electrical properties of an insulating system, the electrical conductivity as a function of the applied DC voltage is normalized to the dependence of conduction current density (*J*) on the applied electric field strength (*E*). In the present study, variation curves (*J*–*E* curves) of the increasing *J* with *E* are used to investigate charge transport characteristics for different semiconductor electrodes, which is formularized by

(1) $$J=A\cdot E^{K}$$

and taking into logarithm form as

(2) lgJ=lgA+K⋅lgE,

where *A* represents a factor relative to the tested dielectric material, and the gradient *K* of lg*J*−lg*E*, which we call nonlinear coefficient, indicates the nonlinearity of electrical conductivity.

**Figure 4:**
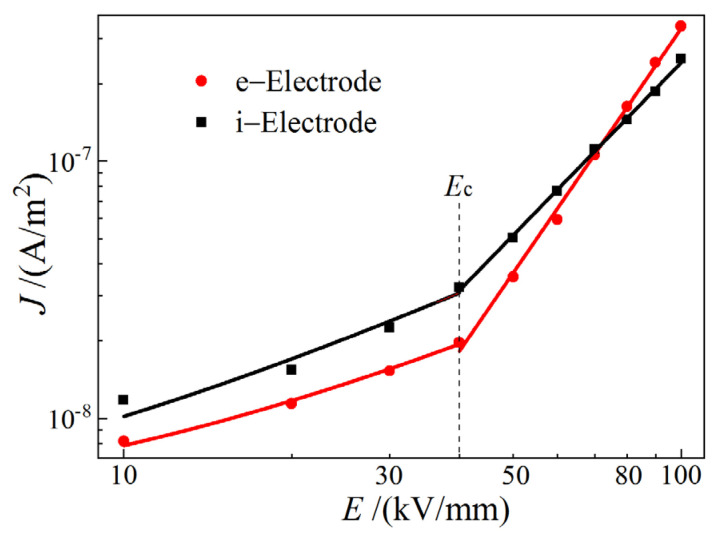
Conduction current density–applied electric field strength (*J*–*E*) varying characteristics in logarithm coordinates tested by i-electrode and e-electrode.

Conforming to the gradient variation of *J*–*E* curves in logarithmic coordinates, as shown in Figure 4, the electrical conductance characteristics can be demarcated into two different carrier transport mechanisms, the critical point of which represents that the dominant electrical conduction altering from Ohmic conductance to space charge limited conductance (SCLC) [28,29]. The critical point *E*_c_ of the electric field at which the evident change of nonlinear coefficient from *K*_1_ to *K*_2_ can be achieved by the piecewise linear fitting for logarithmic *J*–*E* curves, as listed in Table 2.

In the low electric field region (*E* < *E*_c_), the electrical conduction of *K*_1_ < 1 complies with Ohmic conductance mechanism that charge carriers are produced by impurity ionization inside PET material. The higher *J* for using i-electrode than using e-electrode is attributed to the more charge carriers of holes or ions induced in PET from electrochemical reactions at electrode/electrolyte interface of i-electrode. In contrast, under high field *E* > *E*_c_, carriers are injected from electrodes through a Schottky barrier, which is impeded by space charge accumulations near electrodes, resulting in power (*K*_2_ > 2) function of current with electric field (SCLC) that depends on the density and level distribution of charge traps and the carrier mobility. Therefore, the larger *K*_2_ SCLC of transporting electrons injected from e-electrode than that of hole transport from the i-electrode injection can be attributed to the higher trap density (especially for the shallow traps with high capturing section and probability, as shown in TSC spectra of Figure 3a) and smaller effective mass of electrons than those of holes [30]. Even though the current density produced by injecting holes with i-electrode is higher than that with e-electrode under low electric field strength, the larger *K*_2_ leads to a higher *J* of SCLC for e-electrode than i-electrode when the electric field strength is increased higher than 70 kV/mm, as shown by the crossing point of two logarithm *J*–*E* lines in SCLC. The nonlinear coefficient discrepancy caused by using different semiconductor electrodes is another manifestation that the charge injections of electrons and holes can be effectively distinguished by individually using e-electrode and i-electrode, as consistently demonstrated by TSC analyses.

### 3.3. Dielectric Breakdown Strength

**Figure 5:**
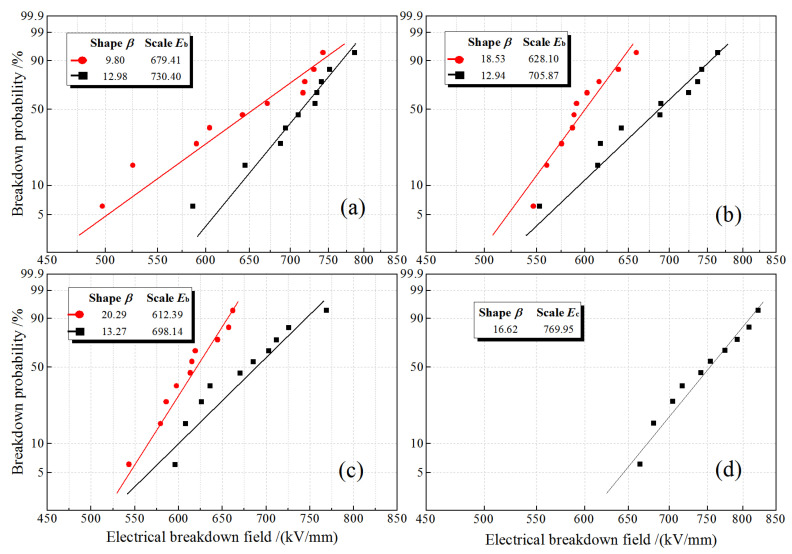
Electrical breakdown fields under DC voltage fitted with two-parameter Weibull statistics for the PET film samples after being applied with DC electric fields of (**a**) 25 kV/mm, (**b**) 75 kV/mm, and (**c**) 100 kV/mm using e-electrode (red sphere) or i-electrode (black square), and (**d**) without pretreatment of applying voltage.

After charge injections respectively with e-electrode and i-electrode by individually applying DC electric fields of 25 kV/mm, 75 kV/mm, and 100 kV/mm for 30 min, the electrical breakdown fields (dielectric breakdown strength, DBS) of PET samples are measured under DC voltage, the results of which are analyzed with two-parameter Weibull statistics, as shown in Figure 5a–c, compared with Figure 5d of the DBS results without pretreatment of charge injection by semiconductor electrode. Scale parameter *E*_b_ (characteristic breakdown field) identifies the breakdown field with a 63.2% probability of dielectric failure, while shape parameter *β* indicates the data dispersivity of DBS experimental results. Due to the local electric field distortion caused by space charge injection, the *E*_b_ tested after charge injection by semiconductor electrode decreases with the increase of charge injection field and persists in a lower value than that from DBS experiments without charge injection pretreatment using semiconductor electrode. The characteristic breakdown fields *E*_b_ of the samples pretreated by the charge injection with i-electrode is generally higher than that with traditional e-electrode.

Shape parameter *β* of DBS Weibull distribution reflects the dispersion of breakdown voltages and can be used to evaluate the energy level distribution and space density of charge traps caused by defects and polar-groups inside insulating materials. In particular, the *β* for charge injections with i-electrode under 25 kV/mm electric field has been significantly improved in comparison to e-electrode, implying a more concentrated distribution of electrical breakdown fields and higher insulation stability has been achieved by using i-electrode, which benefit insulation designs. The DBS results are exactly consistent with the charge injection characteristics from TSC tests that different types of space charges can be discriminated out by utilizing e-electrode and i-electrode for electron and hole injections, respectively. In addition, with the increase of electric field for charge injection pretreatment, DBS shape parameters for using e-electrode and i-electrode magnify remarkably and retain a stable value, respectively, implying that space charges are gradually increased by continuous electron injection into electron traps with e-electrode, as comparatively illustrated in Figure 5a–d. Meanwhile, the low density of hole traps in PET material cannot accommodate the increasing hole injection by i-electrode to be saturated in space charge accumulation, as consistently shown by the peak intensities of TSC spectra in Figure 3a. It is hereby suggested that charge injections will be effectively inhibited by using i-electrode.

### 3.4. Dielectric Frequency Spectrum

**Figure 6:**
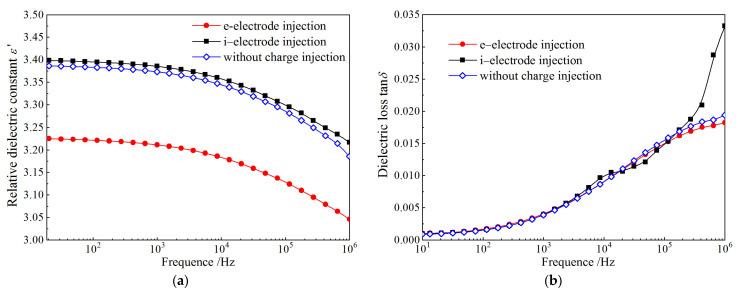
(**a**) Relative dielectric constant and (**b**) dielectric loss for PET film samples after charge injections by applying an electric field of 100 kV/mm individually with e-electrode and i-electrode, compared with the normal sample without charge injection using semiconductor electrode.

After charge injections by applying an electric field of 100 kV/mm with e-electrode and i-electrode, the frequency response of dielectric behavior is tested for PET film samples in comparison to that without pretreatment of charge injection (normal sample), as shown by complex dielectric spectra in Figure 6. Relative dielectric constant *ε*_r_ and dielectric loss tan*δ* spectra of the samples with charge injection pretreatment adopting i-electrode are almost identical to that of the normal sample, which verifies that the charge injection by applying voltage with i-electrode will not cause deterioration in dielectric performances. In contrast, after injecting electrons with e-electrode, the remarkable reduction of dielectric permittivity indicates that dipole orientation polarization has been evidently inhibited by space charges of trapping electrons. In contrast, the sharp increment of dielectric loss at a frequency higher than 3 × 10^5^ Hz for i-electrode originates from the dielectric relaxations of interface charges produced between electrode material and electrolyte, by which i-electrode can specifically impede electron injections.

In comparison to the charge injections with the two kinds of semiconductor electrodes, the effect of space charges on molecular motions of polar dielectrics can be evaluated by the two fundamental dimensionless parameters of *ε*_r_ and tan*δ*. As indicated in Figure 6, *ε*_r_ decreases with the increase of frequency, while tan*δ* shows an upward trend at the frequencies of <10^6^ Hz, which complies with Debye relaxation theory of polar molecules at low frequency. It is noted that the *ε*_r_ for e-electrode declines with frequency a little faster than that for i-electrode due to the fact that the injected electrons have limited orientations and relaxations of polar molecules.

## 4. Conclusions

TSC experiments and first-principles electronic-state calculations demonstrate consistently that the charge carriers of electron and hole can be distinctively injected in the PET paradigm of dielectrics by utilizing e-electrode and i-electrode, respectively. It is suggested that TSC method could be developed into characterizing the energy level distributions of electron- and hole-traps by employing the two kinds of semiconductor electrodes, respectively. TSC peaks arising from the charge injection by i-electrode are significantly lower than that by e-electrode, implying that i-electrode could be used to effectively inhibit electron injections and space charge accumulations.

It is manifested by the fractional power laws of electric current varying with electric-field strength that electrical conductance under low electric-field strength is dominated by internal Ohmic transports. In contrast, electrical transport complies with SCLC mechanism under high electric-fields, which depends on trap characteristics and carrier mobility. Therefore, the larger SCLC nonlinear coefficient of electronic conduction than that of hole conduction can be attributed to the higher trap density and mobility of electrons than that of holes, which confirms that the electron and hole injections can be distinguished by using e-electrode and i-electrode, respectively.

Compared with e-electrode, the dielectric breakdown strength evaluated after the i-electrode charge injection is increased by 1⁄4 with an alleviated dispersion of electrical breakdown fields, indicating that electrons have been effectively restrained to be injected from i-electrode. PET samples pretreated by the charge injection with i-electrode represent a slightly higher dielectric permittivity than that with e-electrode, while showing a significant exacerbation of dielectric loss at high frequencies. The i-electrode inhibition on electron injections can avert the abatement of dielectric permittivity caused by space charge accumulations but will lead to superfluous high-frequent dielectric losses due to the dielectric relaxations of electrode/electrolyte interface dipoles.

## Figures and Tables

**Table 1:** Trap energy level distributions in PET for charge injections individually with different semiconductor electrodes under various applied electric fields.

Charge Injection		Peak/pA at Temperature/°C	Trap Level Depth/eV	Trap Density/(10^20^·eV^−1^·m^−3^)
e-electrode	25 kV/mm	2.7 at 102	1.08	2.21
	50 kV/mm	6.6 at 105	1.09	5.41
	75 kV/mm	11.2 at 39; 23.1 at 105	0.84; 1.09	9.19; 18.95
	100 kV/mm	19.8 at 38; 34.5 at 105	0.83; 1.09	16.24; 28.30
i-electrode	25 kV/mm	-	-	-
	50 kV/mm	3.6 at 90	0.97	2.95
	75 kV/mm	3.8 at 91	0.98	3.11
	100 kV/mm	3.9 at 63; 15.8 at 90	0.92; 0.97	3.20; 12.96
First-principles calculation		-	Electron: 1.12, 1.85, 2.99	-
			Hole: 0.94	

**Table 2:** Critical point *E*_c_, and nonlinear coefficients *K*_1_ and *K*_2_ under the applied electric fields lower and higher than *E*_c_, respectively, for *J*–*E* curves tested with i-electrode and e-electrode.

Electrodes	*E*_c_/(kV/mm)	*K*_1_	*K*_2_
Electronic	39.7	0.63	3.15
Ionic	40.9	0.71	2.23

## Data Availability

Experimental methods and results are available from the authors.
